# Self-Assembled Fullerene Crystals as Excellent Aromatic Vapor Sensors

**DOI:** 10.3390/s19020267

**Published:** 2019-01-11

**Authors:** Natsumi Furuuchi, Rekha Goswami Shrestha, Yuji Yamashita, Tetsuji Hirao, Katsuhiko Ariga, Lok Kumar Shrestha

**Affiliations:** 1 Graduate School of Pharmaceutical Science, Chiba Institute of Science, 15-8 Shiomi-cho, Choshi-shi, Chiba 288-0025, Japan; pm16p04@cis.ac.jp (N.F.); yyamashita@cis.ac.jp (Y.Y.); thirao@cis.ac.jp (T.H.); 2 International Center for Materials Nanoarchitectonics (WPI-MANA), National Institute for Materials Science (NIMS), 1-1 Namiki, Ibaraki Tsukuba 305-0044, Japan; rekhashrestha3@hotmail.com (R.G.S.); ARIGA.Katsuhiko@nims.go.jp (K.A.); 3 Graduate School of Frontier Science, The University of Tokyo, Kashiwa, Chiba 277-0827, Japan

**Keywords:** fullerene crystals, self-assembly, liquid-liquid interface, aromatic vapors, quartz crystal microbalance, sensing

## Abstract

Here we report the aromatic vapor sensing performance of bitter melon shaped nanoporous fullerene C_60_ crystals that are self-assembled at a liquid-liquid interface between isopropyl alcohol and C_60_ solution in dodecylbenzene at 25 °C. Average length and center diameter of the crystals were ca. 10 μm and ~2 μm, respectively. Powder X-ray diffraction pattern (pXRD) confirmed a face-centered cubic (*fcc*) structure with cell dimension ca. *a* = 1.4272 nm, and *V* = 2.907 nm^3^, which is similar to that of the pristine fullerene C_60_. Transmission electron microscopy (TEM) confirmed the presence of a nanoporous structure. Quartz crystal microbalance (QCM) results showed that the bitter melon shaped nanoporous C_60_ performs as an excellent sensing system, particularly for aromatic vapors, due to their easy diffusion through the porous architecture and strong *π–π* interactions with the *sp*^2^-carbon.

## 1. Introduction

Fullerene (C_60_) is a truncated icosahedron (*I*_h_) consisting of purely carbon atoms positioned at the junction of a series of hexagons (20) and pentagons (12) arranged in a cage lattice (diameter ~0.8 nm) and defined by alternating single and double bonds. Due to its excellent redox, optical and optoelectronic properties [1,2,3,4,5], it is an extensively studied nanomaterial in the family of carbon allotropes. After the discovery of fullerene (C_60_) in 1985 substantial advances in fullerene chemistry lead to a number of interesting developments such as carbon nanotubes (CNTs) and graphene [6,7]. Due to energetically unstable double bonds within the pentagon rings, fullerenes display electron accepting properties, i.e., fullerenes are highly electron deficient molecules. Additionally, reactivity of the fullerene molecules increases due to the curvature induced by the cage structure that increases the energy associated with the double bonds. It has been shown that fullerene C_60_ molecules polymerize at high temperature and pressure and also under exposure to ultraviolet (UV) light [8,9]. 

Production of functional materials from molecular units requires essential contributions from nanotechnology such as self-assembly/or self-organization, supramolecular assembly, nanomaterials fabrication or nanobiotechnology [10,11,12,13,14,15]. Supramolecular assemblies of fullerene (C_60_) in the bulk phase or at solid substrate using *π*-stacking interactions enables the production of shape-controlled nano- micro size objects. Well-ordered one-dimensional (1D), two-dimensional (2D) or three-dimensional (3D) forms of fullerene promote the electronic and optical properties, which are essential to design advanced devices or functional systems [16,17,18]. Therefore, studies of the self-assembly of fullerenes in well-defined shape continue to grow [19,20]. 

Several methods such as slow evaporation, vapor deposition, precipitation or re-precipitation and template methods have been proposed for designing the shape-controlled crystalline fullerene objects [21,22,23,24,25,26]. It should be noted that a particular method generally produces a specific morphology. For example, slow evaporation of fullerene solutions in good solvents yields 1D nanostructures. In this method, the aspect ratio of 1D nanostructure can be controlled by the rate of evaporation, temperature and concentration of fullerene. The second method includes vapor deposition on solid substrate, which commonly gives 2D sheet or thin films. Soft- or hard templating methods have also been proposed to design crystalline fullerene materials. Precipitation or re-precipitation methods generally produce 1D rods or tubes and sometimes aggregates with unique fullerene crystal shapes. However, the liquid-liquid interfacial precipitation (LLIP) method developed by Miyazawa and coworkers [27] produces low to higher dimensional fullerene nanostructures. Fullerene crystals of a wide variety of morphologies have been produced using this method. In the LLIP method, alcohol (the antisolvent of fullerene) is slowly added into a fullerene solution in a good solvent (generally aromatic solvents in which fullerenes are soluble) to form a clear liquid-liquid interface [28,29,30,31]. With time alcohol molecules diffuse down towards the good solvent side (alcohols mix well with good solvents) causing an unsaturation at the liquid-liquid interface and as a result crystal nuclei are formed, which will grow further upon diffusion of fullerene from the bulk phase to the interface. The crystal formation mechanism is therefore driven by supersaturation related to the low solubility of fullerene in alcohol, which indicates the importance of the solubility difference of fullerene in the antisolvent and solvent. Needle-like crystals grown in arbitrary directions were observed through the LLIP process at the interface of C_60_ solution in toluene (saturated) and isopropyl alcohol (IPA). Under certain condition, the diameter of the 1D structure was ca. few hundreds of nanometers while lengths were in the hundreds of microns range. Such high aspect ratio 1D nanostructures of fullerene are referred to as fullerene nanowhiskers. Note that contrary to the CNTs, fullerene nanowhiskers do not contain any long-range hollow spaces [32]. Instead, fullerene nanowhiskers are composed of merely fullerene molecules bonded through a combination of Van der Waals interactions and/or chemical bonds (if polymerized). Therefore, they are more bio-friendly and biocompatible. The LLIP method could also be extended to design microporous/mesoporous nanowhiskers or hexagonal nano-/micro-sheets [29,33]. Masuhara et al. [34] have demonstrated fine C_60_ crystals with tunable shape and size by subtle changes in the antisolvent and solvent mixing ratio and temperature. Similarly, Jeong et al. [35] confirmed the multidimensional morphologies of C_60_ microcrystals depending on the type of antisolvent (alcohol type). We have also explored the effect of surfactants on the self-assembly of fullerene at the liquid-liquid interface and found that surfactants, due to their surface-active properties, indeed alter the morphology of the assembled crystals. For instance, in the absence of surfactant 1D C_60_ nanowhiskers (nanorods) and C_60_ nanotubes (diameter 400 nm–2 μm and length 5–20 μm) are obtained from a butanol/benzene system. However, when 0.01% diglycerol monolaurate nonionic surfactant is incorporated into the butanol, flower-like microcrystals with average sizes in the range of 10–35 μm consisting of C_60_ nanotubes are observed [36]. Similarly, C_60_ crystals synthesized at a liquid-liquid interface comprised of IPA and C_60_ solution in ethylbenzene (saturated) exhibited 1D morphology with well-defined faceted structures with average length and diameters of ca. 4.8 μm and 747 nm, respectively. X-ray diffraction patterns confirmed a hexagonal-close packed (*hcp*) structure with cell dimensions ca. *a* = 2.394 nm, and *c* = 1.388 nm. However, interestingly the 1D rod morphology of C_60_ crystals was transformed into “Konpeito candy” type crystals (average diameter ca. 1.2 μm) when the C_60_ crystals were grown in the presence of diglycerol monolaurate surfactant [37]. Not only the morphology was affected as the surfactant also caused a structural transformation from *hcp* to the face-centered cubic (*fcc*) crystal phase with cell dimensions of ca. *a* = 1.4309 nm. 

Using the LLIP method we succeeded at fabricating complex 3D network structures or hierarchical superstructures [38,39]. For example, cube-shape C_60_-fullerene-Ag(I) organometallic heteronanostructures undergo an irreversible structural rearrangement upon exposure to low molecular weight alcohols resulting in the formation of cube-shaped arrays of fullerene nanorods which mimic the original cubic crystal morphology and internal structure [40]. Another interesting example includes 3D cubic-shape assembly of fullerene C_70_ molecules, which could be formed using ultrasonic–agitated liquid–liquid interfacial precipitation between a mesitylene solution of C_70_ and *tert*-butyl alcohol (TBA). Upon exposure to IPA at ambient condition, the C_70_ cubic assemblies were converted into a hierarchical structure of needles protruding out of the cube [41]. Interestingly, the needle-like 1D crystals possess nanoporous structures. These hierarchical nanostructures are advantageous due to their high surface areas, synergistic interactions and multiple functionalities, which are not possible to achieve with conventional materials. Self-assembled fullerene or other nanomaterials have attracted much attention as new materials because of their excellent properties and functions, which largely depend on the size, morphology, surface properties and hierarchy [42,43,44,45]. Note that insertion of micro- or mesopore structures in the fullerene crystals increases the specific surface area and porosity drastically. Such porous fullerene crystals are essentially required in applications like volatile organic compound (VOC) sensing and electrical double layer supercapacitors. However, despite a huge potential applications in energy storage and sensing, nanoporous (micro/mesoporous) fullerene crystals have not well explored [46,47]. 

In this contribution, we report the assembly of C_60_ into unique bitter melon shaped nanoporous crystals. This unique C_60_ crystal was produced by a modified liquid-liquid interfacial precipitation (LLIP) method at 25 °C. Due to a well-developed nanoporous structure the bitter melon shape C_60_ crystals showed excellent vapor sensing performance selective towards aromatic vapors favored by easy diffusion through the porous architecture and strong *π–π* interactions with the *sp*^2^-carbon. We believe hierarchical micro/mesoporous fullerene-based nanomaterial would be an asset to the sensing technology, particularly to sense aromatic guest molecules. 

## 2. Materials and Methods

### 2.1. Materials

Pristine fullerene C_60_ (pC_60_) powder (purity 99.9%) was purchased from MTR Ltd., Cleveland, OH, USA. Isopropyl alcohol (IPA) of purity >99% was purchased from the Wako Chemical Corporation, Tokyo, Japan. Dodecyl benzene purity >99% was a product of Tokyo Chemical Industry, Tokyo, Japan. All these products were used as received.

### 2.2. Self-Assembly of Fullerene C_60_ at Liquid-Liquid Interface

Fullerene C_60_ crystals were synthesized using a modified liquid-liquid interfacial precipitation (LLIP) method. In a typical crystals synthesis, isopropyl alcohol (IPA: 5 mL) was slowly added into a C_60_ solution in dodecylbenzene (1 mL: 1 mg/mL) so that a clear liquid-liquid interface is formed between the two solutions. The mixture was incubated at 25 °C for about 30 min avoiding any external mechanical disturbances. The mixture was then sonicated for 5 min in a bath sonicator 3510 BRANSON (Gaithersburg, MD, USA) at 100 W followed by vigorous vortex mixing for 1 min. Finally the mixture was incubated at 25 °C for 24 h. The precipitates (self-assembled crystals) were collected by centrifugation and denoted as ‘as prepared’ sample. The as prepared crystals were washed with excess of IPA (10 mL) and dried in vacuum at 80 °C for further characterizations. 

### 2.3. Characterizations

The prepared self-assembled fullerene nanomaterials (before and IPA after washing) were characterized by powder X-ray diffraction (RINT2000 diffractometer, Rigaku, Tokyo, Japan, with Cu-K_α_ radiation (*λ* = 0.1541 nm, scanning electron microscopy (SEM: Model SU-8000, Hitachi Tokyo, Japan) which was operated at 10 kV), transmission electron microscopy (TEM, Model JEM-2100F operated at 200 kV, JEOL, Tokyo, Japan), Raman scattering spectroscopy (T64000, Jobin-Yvon Edison, Edison, NJ, USA) at a wavelength of 514.5 nm and Fourier transform infrared (FT-IR) spectroscopy (model 4700 instrument, Nicolet, Walthan, MA, USA). For the preparation of SEM samples, a dilute suspensions of fullerene crystals in IPA were drop casted on a clean silicon wafer and dried at 80 °C for 6 h. All the SEM samples were coated with platinum (~2 nm) by sputtering using a Hitachi S-2030 ion coater. TEM samples were prepared by dropping suspensions of fullerene crystal onto standard carbon-coated copper grids. TEM samples were dried at 80 °C in vacuum for 24 h.

### 2.4. Vapor Sensing by Quartz Crystal Microbalance (QCM)

Quartz crystal microbalance (QCM) is a powerful technique for detection of small changes in mass during thin film deposition, gas adsorption or desorption and structural changes. In the present study, we used a resonance frequency of 9 MHz (AT-cut) and the frequency change of the Au-resonator coated with our material (QCM electrode) was measured during adsorption of vapor phase. Frequency of the QCM electrode in air was stable within ± 2 Hz over more than 10 min. QCM sensor electrode was prepared as follows. Bitter melon shape C_60_ crystals (0.5 mg) were dispersed in ethanol (1 mL) and vortex shaken for 30 s. Note that dispersion in ethanol did not change the morphology of the prepared crystals. 2 µL of the resulting suspension was then drop casted onto the QCM electrode (Au resonator). The electrode was dried at 80 °C in vacuum for 12 h. The modified QCM electrode was then set in the instrument and exposed to different solvents vapors (10 mL) at 25 °C in a sealed chamber to prevent the escape of vapors during the adsorption measurements, i.e., the atmosphere is saturated with the solvent vapors. Once the frequency change reached equilibrium, the electrode was exposed to air to desorb the vapor. Repeatability of the QCM electrode was also tested by recording the time dependence of frequency shift (Δ*f*) during alternate exposure and removal of the solvent vapors. VOC sensing performance of the bitter-melon shape nanoporous fullerene crystals was also compared with pristine C_60_ and as prepared crystal without nanopores before IPA washing. For this purpose, QCM electrodes were prepared following the similar method so that the mass of active materials loaded are essentially the same (1 μg).

The change in mass m (g cm^−2^) of the sample deposited on the QCM electrode can be measured by the oscillating frequency of the quartz electrode. The frequency change (Δ*f*) corresponds to amount of sample loaded on the QCM electrode, which can be calculated from the Sauerbrey equation:

(1) $$\Delta f=\left(\frac{2f_{0}^{2}}{A\sqrt{\rho_{Q}\mu_{Q}}}\Delta m\right)$$

where ƒ_0_ (Hz) is the natural frequency of the quartz crystal, Δ*m* is mass change (g), A is area between electrodes (cm^2^), *ρ*_Q_ is quartz density (2.649 g cm^−3^) and *µ*_Q_ is the shear modulus (2.947 × 10^11^ g cm^−1^ s^2^).

## 3. Results

**Figure 1:**
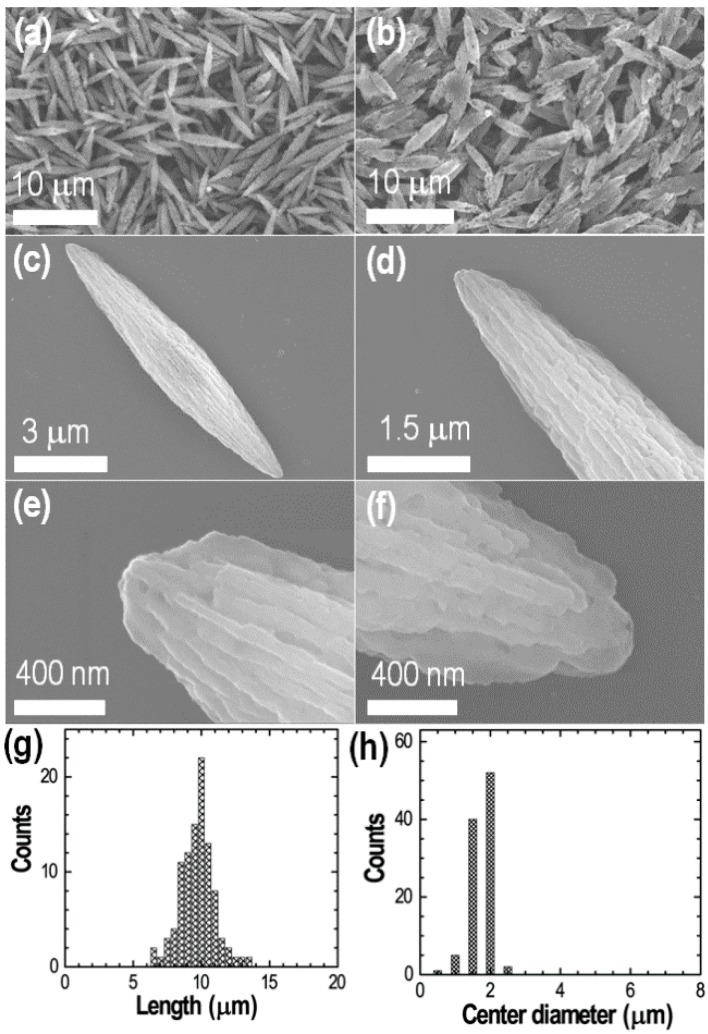
SEM images of fullerene C_60_ crystals self-assembled at a liquid-liquid interface of IPA and C_60_ solution in dodecylbenzene at 25 °C: (**a**,**b**) Low magnification images showing over all surface morphology; (**c**,**d**) SEM images of one crystal showing bitter-melon shape; (**e**,**f**) High resolution SEM images; (**g**,**h**) Histograms of length and diameter distributions.

The surface morphology of self-assembled fullerene C_60_ crystals synthesized at 25 °C using the modified liquid-liquid interfacial precipitation (LLIP) method at an interface of isopropyl alcohol (IPA: 5 mL) and C_60_ solution in dodecylbenzene (1 mL: 1 mg/mL) was studied by scanning electron microscopy (SEM). SEM images of both the samples (as prepared crystals before and after IPA washing) were taken. SEM observations revealed the unique assembly fullerene C_60_ into bitter melon shaped crystals (Figure 1a–f). The as-prepared crystals before washing appear in aggregated form (Figure S1: see Electronic Supporting Information). Washing with IPA caused segregation of the crystals. High resolution SEM image shows that the individual crystal possesses a rough surface along its entire length. Figure 1g,h shows histograms of length and diameter distributions of randomly selected 100 crystals. Note that the diameter of the crystal is not uniform along the axial length. The diameter in the histogram is referred to the center diameter. a histogram of the length distribution shows that lengths of crystals are in the range of ~6.5 to 13.5 μm with majority of crystal exhibiting lengths of ~10 μm (Figure 1g). The histogram of center diameters suggests a mean diameter of 2 μm (Figure 1h). SEM images also show that some of the crystals are interconnected into different shapes such as T-shapes, Y-shapes, and star-shapes, indicating the anisotropic growth of the crystals (Figure S2). 

**Figure 2:**
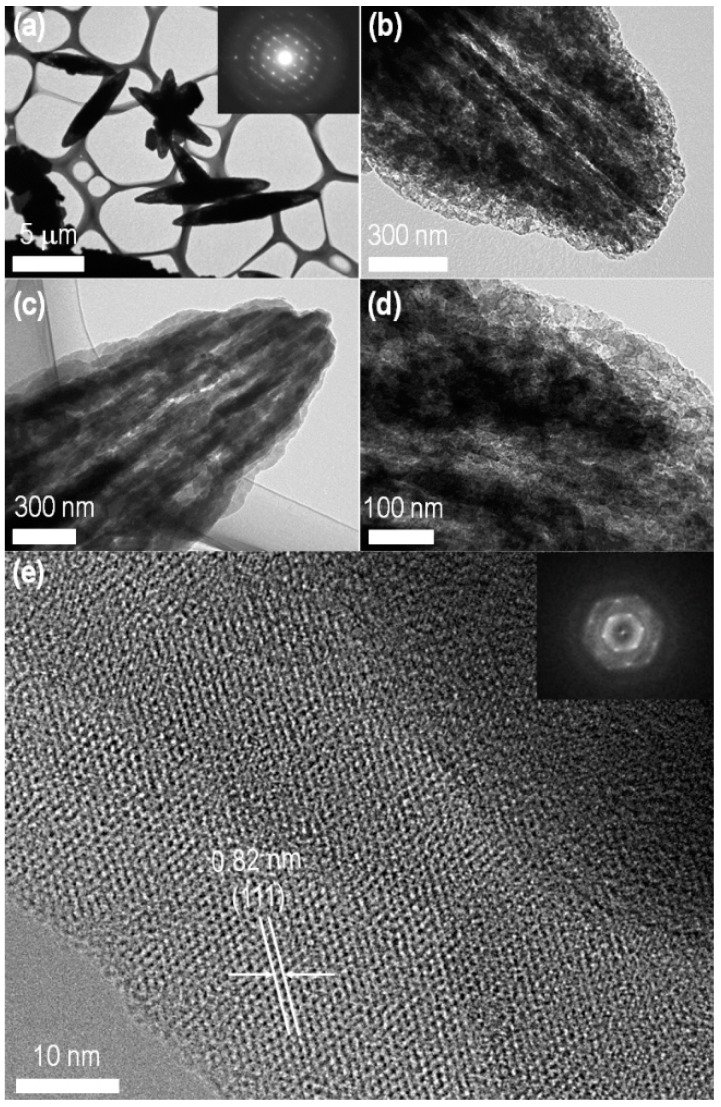
Transmission microscopic images of C_60_ crystals prepared a liquid-liquid interface of IPA and C_60_ solution in dodecylbenzene at 25 °C: (**a**–**d**) Low magnification TEM images; (**e**) HR-TEM image. Insets of panel “a” and “e” represent SAED patterns.

The crystal structure of the assembled fullerene C_60_ was further characterized by transmission electron microscopy. As revealed by SEM, the bitter melon shape of the crystals is further confirmed by TEM (Figure 2a). Some of the assembled structures are interconnected with crystalline nature as confirmed by the spot-type selected area electron diffraction pattern (inset of Figure 2a). High magnification TEM images revealed the bitter melon shape crystals exhibit nanoporous structures (Figure 2c,d). It is interesting to note that the as-prepared crystals before washing do not have such a nanoporous structure (Figure S3) demonstrating that nanopore formation is caused by the IPA washing. Since fullerene C_60_ is partially soluble in IPA (0.0021 mg/mL), surface dissolution of C_60_ takes place upon washing with IPA. A high resolution TEM (HR-TEM) image shows a dense packed lattice fringes of fullerene with lattice spacing is ca. 0.82 nm corresponding to the distance between two (111) planes of the face-centered cubic (*fcc*) phase of the pristine C_60_. 

**Figure 3:**
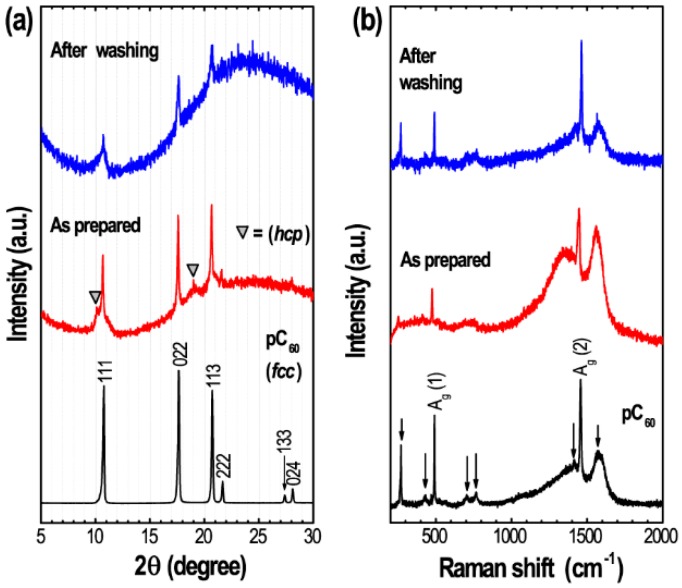
(**a**) Powder X-ray diffraction (pXRD) of pC_60_ and self-assembled bitter-melon shaped fullerene C_60_ crystals before and after washing; (**b**) the corresponding Raman scattering spectra recorded at 25 °C.

The crystal structure of the self-assembled C_60_ is confirmed by powder X-ray diffraction (pXRD) technique. Figure 3 shows diffraction patterns of C_60_ assemblies before and after IPA washing. For comparison, diffraction pattern of pristine C_60_ (pC_60_) is also included. Bitter melon shaped C_60_ assemblies before IPA washing show intense pXRD peaks at diffraction angles of 10.7°, 17.58° and 20.68°corresponding to (111), (022), and (024), planes of *fcc* crystal phase with the cell dimensions ca. *a* = 1.425 nm, and *V* = 2.899 nm^3^.

Weak diffraction peaks at diffraction angles of 10.14° and 19° (as highlighted by symbol) corresponds to (101) and (401) planes of *hcp* crystal phase, which is commonly observed in solid solvates due to the entrapment of solvent molecule at the interstitial site of the cubic lattice during crystallization [48,49]. 

The diffraction peaks corresponding to *hcp* phase disappear upon washing/and drying of the samples and the samples displays pXRD peaks corresponding to *fcc* phase only with cell dimension ca. *a* = 1.427 nm, and *V* = 2.907 nm^3^, which are very close to the cell dimension of pristine C_60_; *a* = 1.421 nm, and *V* = 2.871 nm^3^. Figure 3b shows Raman scattering spectrum of pC_60_ and the prepared C_60_ crystal before and after IPA washing. Raman spectra were recorded upon exciting with a 514.5 nm laser at 25 °C. Raman spectra showed two Ag [A_g_(1), and A_g_(2)] and six H_g_ vibration bands [H_g_(1), H_g_(2), H_g_(3), H_g_(4), H_g_(7) and H_g_(8)]. Out of these Raman active bands, the A_g_(2) band corresponds to pentagonal pinch mode and has been used as an analytical probe for the structural and optoelectronic properties of fullerene C_60_ molecule [50]. For instance, a blue shift of A_g_(2) band is an indication of polymerization of C_60_ molecules. The A_g_(2) Raman band of bitter melon shaped fullerene C_60_ crystal remains essentially unchanged before and after washing and is similar to that of pC_60_. This indicates that molecular C_60_ dominates in the bitter melon shaped nanoporous fullerene C_60_ crystal and laser irradiation during measurements did not cause polymerization of C_60_ molecules, i.e., self-assembled crystals are molecular solid without any chemical bonds between the adjacent C_60_ molecules.

**Figure 4:**
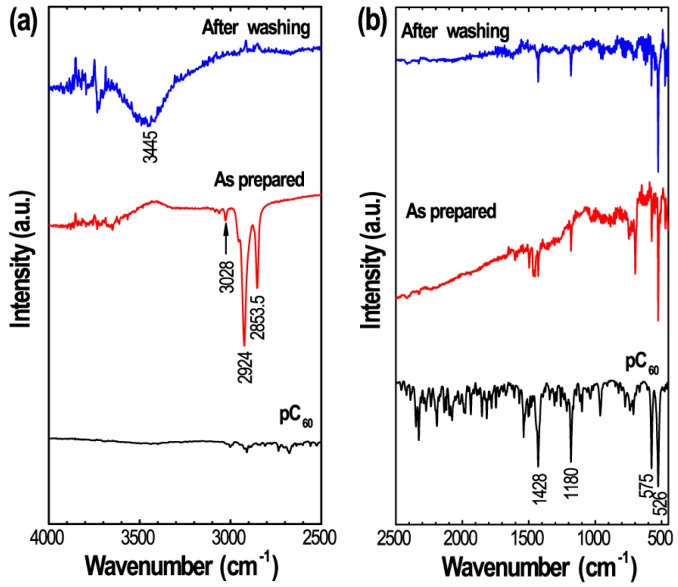
FT-IR spectra of pC_60_ and self-assembled bitter-melon shaped fullerene C_60_ crystals before and after washing measured at 25 °C: (**a**) FT-IR spectra in high wavenumber region; (**b**) Corresponding FT-IR spectra in low wavenumber region.

In order to further confirm the solid solvate structure as revealed by pXRD, we have recorded FT-IR spectra of prepared fullerene C_60_ crystals before and after washing and compared the data with pC_60_. Figure 4 shows FT-IR data recorded at 25 °C in low and high wavenumber regions. In addition to four major peaks at 526, 575, 1180 and 1428 cm^−1^ corresponding to pC_60_, three additional peaks at 3028, 2924 and 2853.5 cm^−1^ that corresponds to aromatic (3028 cm^−1^) and aliphatic (2924 and 2853.5 cm^−1^) C–H stretching can be seen in the FT-IR spectrum of the as-prepared sample before IPA washing, indicating that the crystals contain some dodecylbenzene solvent molecules, i.e., as prepared crystals are C_60_ solid solvates. FT-IR bands corresponding to the C–H stretching disappeared and one additional peak at 3445 cm^–1^ (O–H stretching) appeared the bitter-melon shape crystal after IPA washing.

**Figure 5:**
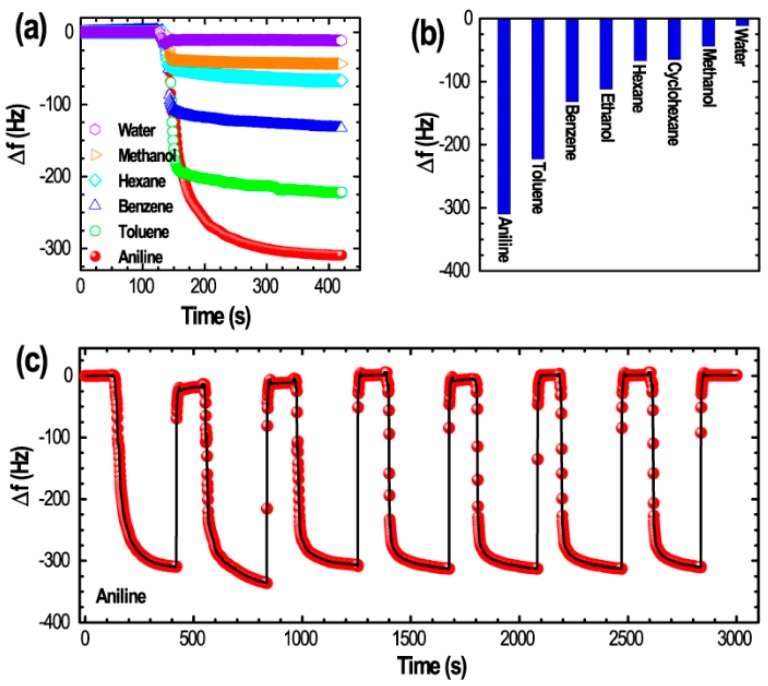
Vapor sensing performance of bitter melon shape nanoporous C_60_ crystals: (**a**) Frequency shifts (Δ*f*) upon exposure of QCM electrode to water, methanol, hexane, benzene, toluene and aniline as typical example; (**b**) Summary of sensor performance, and (**c**) Repeatability test upon exposure and removal of toluene vapors up to seven cycles.

Judging from the surface and structural properties, we anticipated that our nanoporous bittermelon shaped fullerene C_60_ crystals could be suitable candidates for sensing toxic VOCs such as benzene, toluene and aniline, and we have studied the vapor sensing capacities for sensing toxic aromatic solvents using the quartz crystal microbalance (QCM) technique. QCM sensors are favored over other gas analysis techniques such as gas chromatography and mass spectroscopy, as they are compact in size, simple design and provide the real time analysis. Figure 5a shows a typical example of the time dependent frequency shifts (Δ*f*) of the bitter melon shaped nanoporous fullerene C_60_ crystals modified QCM electrode upon exposure to the different organic solvents vapors; methanol, hexane, benzene, toluene and aniline. For comparison, frequency shifts upon exposure of water vapors is also included.

As it can be seen in Figure 5a, the frequency shift is very rapid upon exposure of the QCM sensor to the solvent vapors and when the solvent vapors are removed or air is exposed the frequency goes back to its original position (desorption is also rapid: Figure S4a). Furthermore, the value of frequency shift, which corresponds to the adsorption of gas molecules, strongly depends on the nature of the vapor phase guest molecules. Aromatic solvent vapors caused huge frequency shifts compared to aliphatic hydrocarbons of apparently similar molecular size. Alcohols and water indeed caused only a small change in the frequency (Figure 5b). Frequency shifts caused by the adsorption of aromatic solvent vapors are ca. 310 Hz (aniline), 222 Hz (toluene), 132 Hz (benzene). While the frequency shifts caused by the adsorption of aliphatic solvent vapors are ca. 67 Hz (hexane), 65 Hz (cyclohexane). Frequency shifts caused by the adsorption of ethanol, methanol and water were ca. 111 Hz (ethanol), 43 Hz (methanol) and 11 Hz (water), respectively. QCM results demonstrate that the bitter melon shape nanoporous C_60_ crystals is preferential hosts for aromatic vapors, i.e., they are more selective towards aromatic vapors. The selectivity of QCM sensing decreases in the following order: aniline > toluene > benzene > ethanol > hexane > cyclohexane > methanol > water. We have also tested the repeatability of the QCM electrode upon alternate exposure and removal of aniline vapors (Figure 5c). The electrode showed quite good repeatability without losing sensing performance after seven cycles, demonstrating the potential of the material in QCM device fabrication.

## 4. Discussion

Environmental pollution has become one of the key problems with the advancement of nanotechnology. Air pollutants, such as VOCs and respirable particulate matter (PM_2.5_ and PM_10_) diffuse over long or short distances and contaminate the environment. The air polluted with VOCs has both acute and chronic effects on human health, affecting a number of different systems and organs. This ranges from minor upper respiratory tract irritation to chronic respiratory and heart disease, lung cancer, acute respiratory infections, aggravated pre-existing heart and lung diseases, or asthmatic attacks. Both indoor and ambient air is contaminated with toxic VOCs such as toluene, aniline and benzene due to the use of common household products such as paints, adhesives, synthetic fragrances, and smokes, which are inevitable. Needless to say these VOCs are extremely bad for a healthy environment and life. Therefore, molecular sensing of toxic organic substances has become an important subject and many researchers have been working to design a novel nanomaterials to design functional system to address these environment-related problems [51,52,53,54]. Previous investigations have underlined the fact that a well-designed host nanostructure, particularly high surface area nanoporous materials, are essential for enhancing sensor performance [55,56,57,58,59,60]. 

Micro/mesoporous hierarchical fullerene nanomaterials are anticipated to display higher efficiency as gas sensors, particularly for aromatic vapors due to their unique *π*-conjugated structure, high hydrophobicity and strong van der Waals and *π-π* interactions that contribute greatly to the sensing performance and higher sensing selectivity response to aromatic guests [41,59]. As seen in Figure 5a,b, a higher selectivity towards aromatic solvent vapors compared to the aliphatic hydrocarbon vapors of similar dimensions is caused due to enhanced interaction between *sp*^2^-featured fullerene assembly with aromatic gases, high-contact probability, and easy diffusions of the guest gases through the mesoporous structure of the bitter-melon-shaped fullerene C_60_ crystals. Since the vapor pressure (log(pressure/mmHg)) of aromatic solvents benzene (1.978), toluene (1.462) and aniline (0.489) is lower compared to the aliphatic hydrocarbon, *n*-hexane (2.179), the observed higher sensing selectivity towards toluene or pyridine vapors cannot be due to the difference in saturated vapor pressure. It is caused due to a unique structural feature of our material. Sensing performances of as prepared crystals without nanoporous structure and pC_60_ are very poor compared to the nanoporous crystals (Figure S4b) demonstrating the importance of porous structure, which promotes easy diffusion of aromatic solvent vapors into the mesoporous architecture favored by strong *π-π* interactions between host and guest. 

## 5. Conclusions

We have fabricated self-assembled bitter melon shaped mesoporous C_60_ crystals by a dynamic liquid-liquid interfacial precipitation (LLIP) method at ambient conditions of temperature and pressure. Due to a well-developed mesoporous structure the bitter melon-like fullerene crystals showed excellent vapor sensing performance with higher selectivity towards aromatic vapors. QCM Frequency shifts caused by the adsorption of solvent vapors are ca. 310 Hz (aniline), 222 Hz (toluene), 132 Hz (benzene), 67 Hz (hexane), 65 Hz (cyclohexane), 111 Hz (ethanol), 43 Hz (methanol) and 11 Hz (water) demonstrating that the bitter-melon-shape nanoporous C_60_ crystals are more selective towards aromatic vapors compared to the aliphatic counterparts. The selectivity of QCM sensing decreases in the following order: aniline > toluene > benzene > ethanol > hexane > cyclohexane > methanol > water. We believe that nanoporous fullerene-based nanomaterial would be an asset to the sensing technology, particularly to sense aromatic guest molecules. 

## Supplementary Materials

The following are available online at http://www.mdpi.com/1424-8220/19/2/267/s1, Figure S1: SEM images of as prepared C_60_ crystal before washing: (**a**–**d**) Low magnification SEM images showing the aggregated structure; (**e**,**f**) High magnification SEM images, Figure S2: Additional SEM images of the crystal after washing showing different shapes, Figure S3: TEM images of as prepared crystal before washing: (**a**–**d**) Low magnification TEM images. (**e**) HR-TEM image. Insets of panel “a” and “e” represent SAED patterns, Figure S4: Additional QCM data: (**a**) Frequency shits upon exposure and removal of solvents vapors; (**b**) Comparison of sensing performance of mesoporous bitter-melon-shape crystals (after washing) with as prepared crystal and pristine C_60_.
