# Supplementary material for: Self-Assembled Fullerene Crystals as Excellent Aromatic Vapor Sensors
Source: Sensors (Basel). 2019 Jan 11;19(2):267. doi: 10.3390/s19020267 (PMC6359261; doi:10.3390/s19020267)
Supplement: Supplementary file 1 [file sensors-19-00267-s001.pdf]

sensors

MDPI

*Article*

# Self-Assembled Fullerene Crystals as Excellent Aromatic Vapor Sensors

Natsumi Furuuchi [1], Rekha Goswami Shrestha [2], Yuji Yamashita [1], Tetsuji Hirao [1], Katsuhiko Ariga [2,3] and Lok Kumar Shrestha [2,*]

## 1. SEM images of as-prepared fullerene crystals

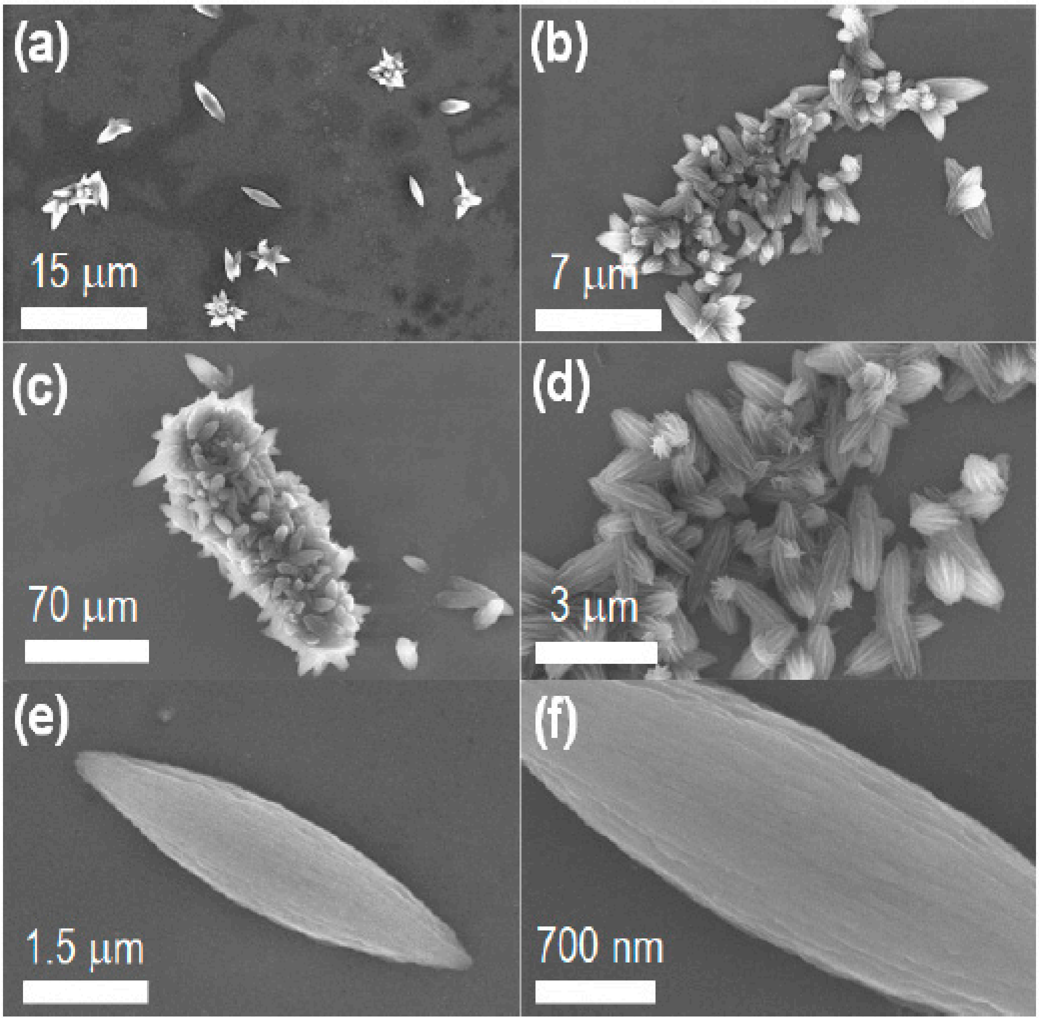


**Figure S1.** SEM images of as prepared C60 crystal before washing: (**a-d**) Low magnification SEM images showing the aggregated structure; (**e-f**) High magnification SEM images.

## 2. Additional SEM images of fullerene crystals after washing

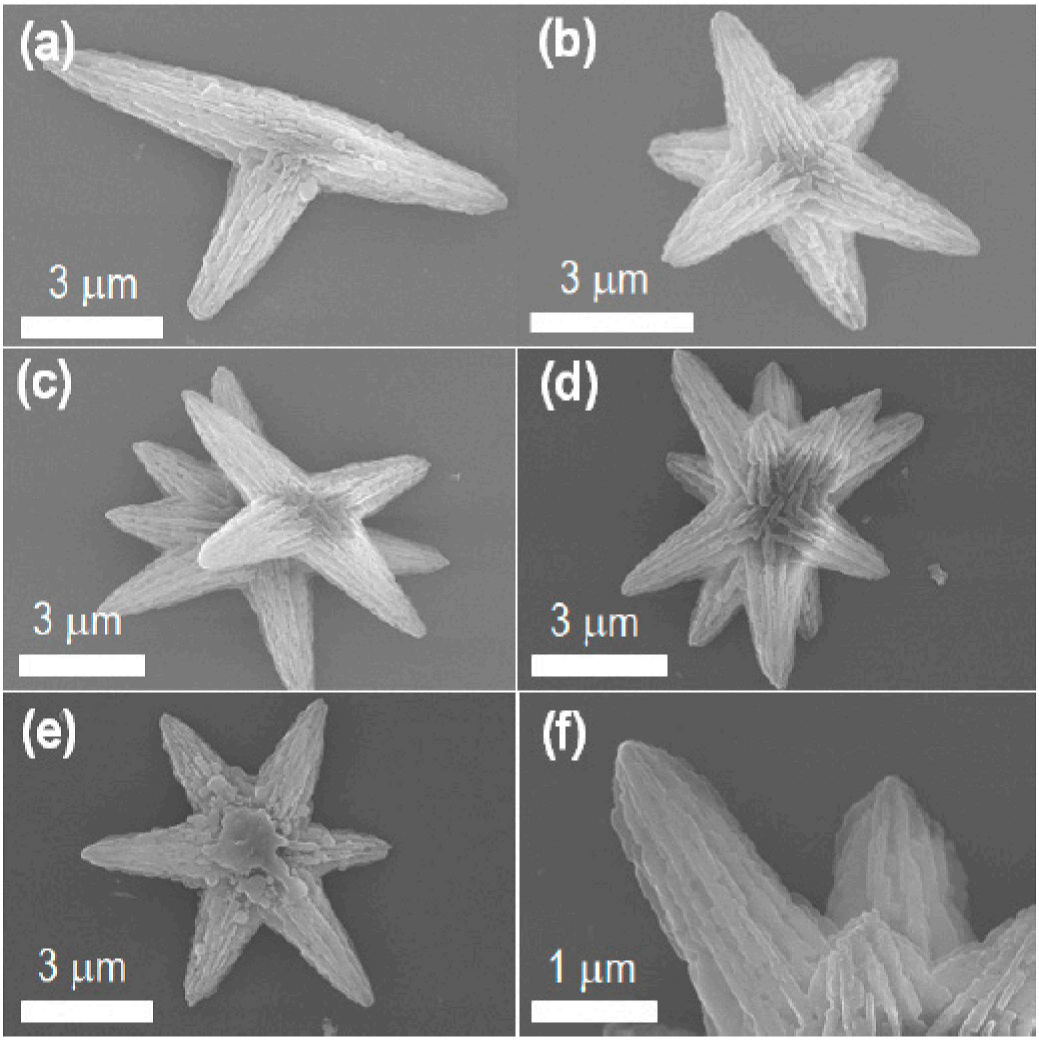


**Figure S2.** Additional SEM images of the crystal after washing showing different shapes.

## 3. TEM images of as-prepared fullerene crystals

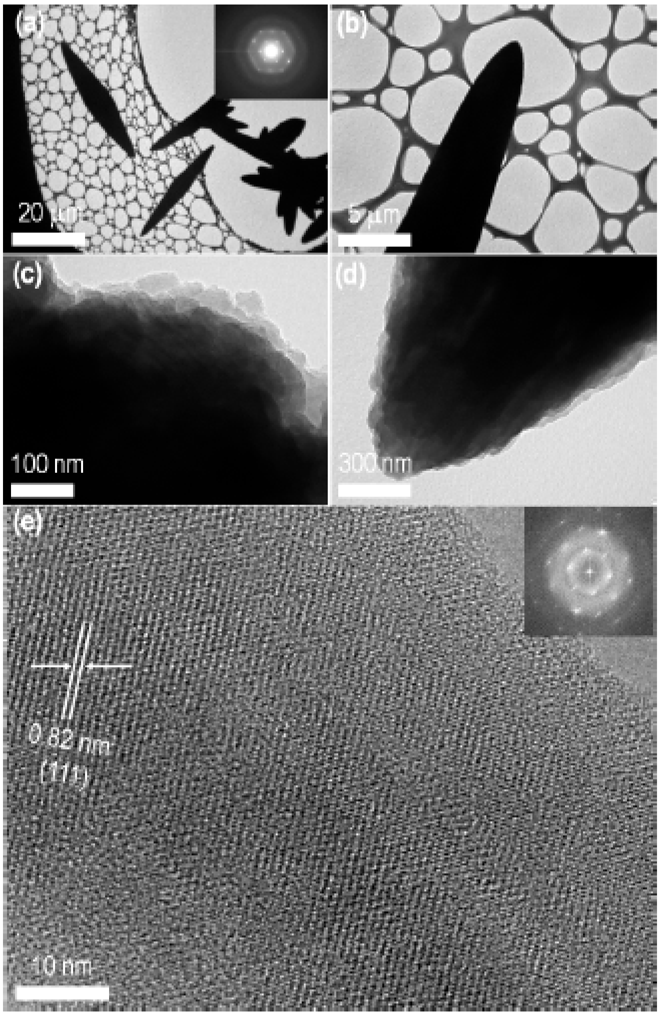


**Figure S3.** TEM images of as prepared crystal before washing: (**a-d**) Low magnification TEM images. (**e**) HR-TEM image. Insets of panel "a" and "e" represent SAED pattern.

## 4. Additional QCM sensing data

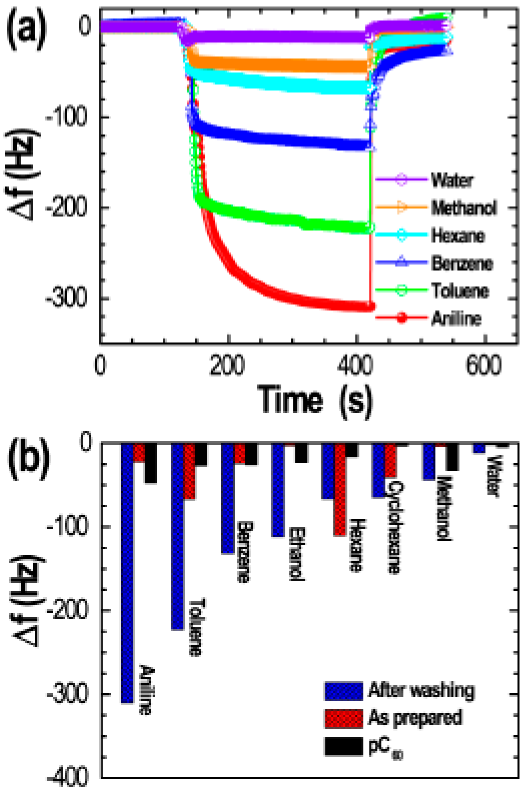


**Figure S4.** Additional QCM data: (**a**) Frequency shits upon exposure and removal of solvents vapors; (**b**) Comparison of sensing performance of mesoporous bitter-melon-shape crystals (after washing) with as prepared crystal and pristine $C_{60}$.
